# Health Related Studyability-An Approach to Structure Health Promotion Interventions at Universities

**DOI:** 10.3389/fpubh.2021.654119

**Published:** 2021-05-12

**Authors:** Mona Kellner, Klaus Weiß, Janina Gassert, Gerhard Huber

**Affiliations:** Institute of Sports and Sports Science, Heidelberg University, Heidelberg, Germany

**Keywords:** studyability, workability, health-related studies, university health promotion, health management

## Abstract

So far, the only existing prerequisite to enter an academic institution is a specific diploma, like a high school diploma, or another comparable certified document. Other requirements may only be a numerus clausus for certain fields of study to pave the bureaucratic way for a prospective student into their university life. The way first year students dive into their first academic experiences is entirely left to themselves. (Soft) Skills and Competences that exceed the expertise of the chosen courses but are essential for this new, and very challenging, chapter of their lives are not taught to them. Therefore, student health promotion for young adults is essential to build and sustain a healthy lifestyle during their academic careers. Nevertheless, it is important to consider not only a student's perspective but also structural and organizational conditions within the academic institutions. The further development of Ilmarinen's concept of workability may help to construct a theoretical and empirically based concept to implement health-promoting conditions for a student health promotion system at universities. Ilmarinen's concept was chosen by the work group in terms of the structure, which may be adapted to a university since it can be seen as a student's workplace.

## Introduction

Student health promotion takes on various tasks. On the one hand, those responsible should ensure that students are and remain in good physical and mental health during their studies. On the other hand, health skills for the professional future are to be taught in order to demonstrate a healthy lifestyle to the target group. The main goal is to reach a health-related studyability – the ability to complete a degree in a physically and mentally healthy manner. Prospectively, this may prepare the workability for the future career.

Current surveys show that even within this rather young and healthy population there may be vulnerable groups who are at risk of serious health consequences. Empirical studies on students in Germany show that we talk about manifold health risks concerning these groups:

- Recurrent (min. once a week) physical complaints like back and neck pain and body aches (1, 2).
- A high occurrence of depressive symptoms, compared to the normal population (1, 3).
- An increased negative experience of stress (3).
- $\frac{3}{4}$ of the target group do not reach the recommendations for physical activity given by the World Health Organization (WHO) (1).
- An increased prescription of antihypertensive and antithrombotic drugs (2).

Analyses show that the population is highly prone to progressive metabolic, orthopedic, and mental health risks. In accordance with the approach of primary prevention and health promotion it is essential to intervene at this point in order to halt this negative development.

Even students who are meant to be healthy for the moment are exposed to these risk factors. Primary preventive action must be taken at this point to ensure that the health status of these young people does not deteriorate dramatically resulting in distal effects later in their working lives.

However, health promotion interventions should not only focus on the behavior of the students. At the same time, it must be determined which conditions at universities have to be addressed to enable healthy studies. For this purpose, an already existing model from workplace health management is to be adapted and enhanced to the students' needs and the university setting. The model ultimately serves as a heuristic, and to identify but also to develop and evaluate measures for areas of action in primary prevention. Moreover, it may be used to adapt the overall structures and conditions.

## House of Workability

The house of workability is a theoretical concept to determine the resources and the needs of employees in a multifunctional context. This model was developed on the basis of empirical findings related to the workability index (WAI) by a work group of the Finnish Institute for Occupational Health (FIOH) around Juhani Ilmarinen (4). “Work ability can be defined as the balance between human resources and the demands of work” (5). This ability is based on various factors, which are influenced not only by the employee but also by the employer/company (4, 6).

The WAI is a construct from the point of view of occupational health. It is used as a predictor for any kind of inability to work, such as sickness absences or early retirements. Thus, it is a well-established assessment tool, which is often used in the workplace setting.

The underlying theoretical model, the house of workability, pursues a less deficit-oriented goal and tries to represent those resources, which are the basics of workability, in a house with several floors. The focus of the model is substantiating the resources and support options to maintain workability.

The configuration of each floor may develop and improve the workability, which is influenced by the following factors (5, 7):

- Ground floor: health and functional capacities: physical, mental and social health, and efficiency in relation to work.
- 1st floor: competences, experiences, learning: technical, methodical, and socially supportive measures to cope with the work tasks.
- 2nd floor: values, attitudes, and motivation: appreciation and fair treatment by the superior, trust in the superior, commitment, and motivation for work.
- 3rd floor: work, work arrangements, work community, leadership: organization of work, support from superiors and colleagues in difficult situations, feedback from superiors.

## Transformation Into The House of Studyability

The original model is perfectly suited to transform it into the house of studyability in terms of the technical and structural requirements and complement it with health-related competences, values, motivation, and structural organization (8). In the end, a concept is being created which is structurally based on the house of workability, but defined through more flexible design criteria that comprise the requirements of an academic institution.

The fact that one's studyability is influenced by multidimensional aspects is important to be acknowledged in the field of student health promotion. Therefore, it is to be underlined, that any intervention concerning health promotion must not only concentrate on the student's behavior, but also on structural, technical, and organizational factors and the working conditions within the academic institution.

The factors functional capacities, competences, values, attitudes and motivation, work community, and leadership are consistent with the original model. Just like the house of workability the house of studyability will distribute that multiple factors influence the accomplishment of the given tasks and need to be seen in a complex interaction.

Since the working conditions, the complex daily tasks, and the functions and hierarchies differ very much in between universities, and even within universities and their faculties, the main focus lies on the adaption of every single floor to the university system in general. Further, adjustments may need to be made for each university referring to some organizational and structural conditions.

## Former Experience and Future Vision of The Project

The department prevention and rehabilitation of the Sports Institute of Heidelberg University has been dealing with the issues of a functional workplace health promotion and its requirements for more than 25 years. The work group was able to give support to several projects and initiatives in a wide variety of industries. Such as highly qualified manufacturing areas in the automotive industry, universities, public service administrations, branches with a high level of physical activity, and small- and medium-sized businesses.

Adding the current research results to this experience, the work group was able to identify conditions and criteria which are decisive as quality standards to establish a successful workplace health management. Therefore, these quality standards are taken into account during the process of the development of the house of studyability.

These are the quality standards mentioned above:

- A situation and needs analysis on a regular basis.
- Any activities and actions will be initiated based on the identified needs.
- A suitable and well-established organization and process structure.
- Activities and interventions take place right at the workplace.
- Development and improvement of self-efficacy and health literacy.
- Evaluation and success control on a regular basis.

These criteria not only stand for a way to success but also for the opportunity to act sustainably.

Since requirements and situations in the social life tend to develop dynamically those quality standards and the strategic approach to move forward should be adapted consecutively. Transferring the ground works of health promotion to academic institutions is obvious since the social potential of the future is formed and shaped in these institutions. Students of today may become the leaders of tomorrow and therefore take on responsibilities for their employees in the future. In this ever-changing world health promotion is an incredibly important topic, which should be internalized as early as possible.

## Discussion and Outlook

Model constructs such as the house of studyability can be used as an orientation in the development of processes and sustainable structures. In the further process, the individual floors of the house of workability will be transferred to the university structures, their functionality will be checked and, if necessary, adapted. For this purpose, we work closely in exchange with the sports institutes of the University of Tübingen and the KIT Karlsruhe and review our results over and over in a critical discussion. In exchange with the working group of the German Network Health Promoting Universities, which is working on a reflection and development tool for the expansion of the health management at universities, further, components of the house of studyability will be added.

The foundation to “furnish” the house has been laid: there are already numerous studies in Germany on the psychosocial health and physical and mental health behavior of students. Instruments for collecting these data are available and are currently being widely used in order to generate a large quantity of data that allows a statement to be made concerning the health situation of students.

However, if one ascends to the higher floors of the building, the data situation becomes thinner. Hardly any survey addresses the competences of students, focuses on the motivation and values that stand between students and those responsible at the university, or questions the organization of work, which has a significant influence on everyday study life. All these factors, however, make a significant contribution to whether a student feels healthy - or not.

**Figure 1:**
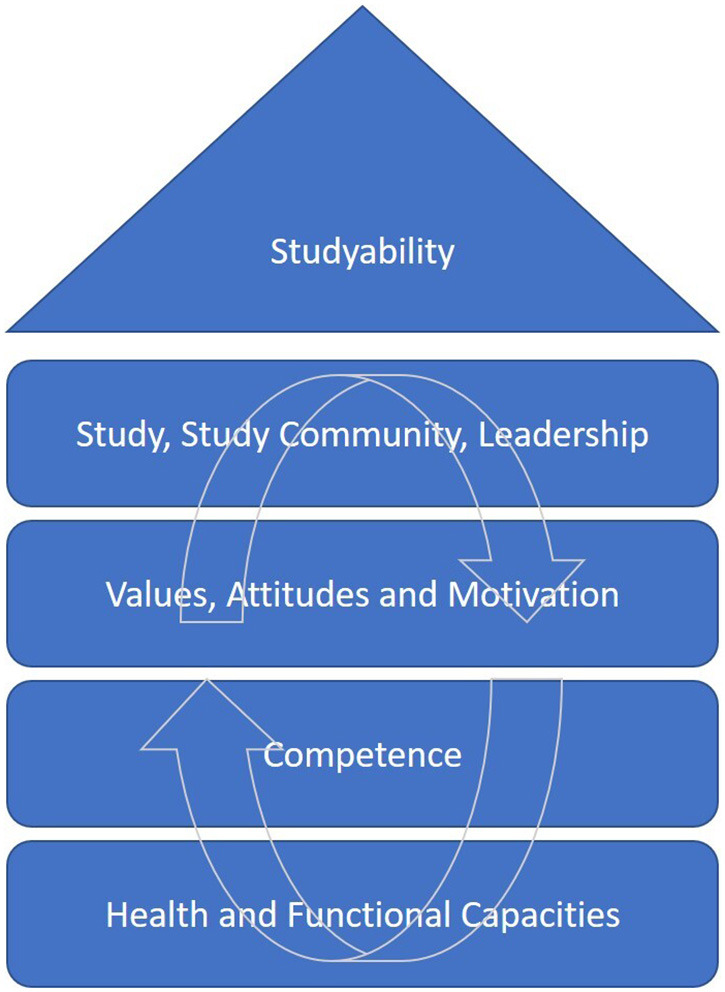
The house of studyability displays the interaction between the factors [adapted from (5)].

Nevertheless, national and international surveys determine a certain vulnerability of the group of students. As described earlier, many students already suffer from physical as well as mental disorders - others pave the way for later developing serious chronic diseases by sitting too long and by being physically inactive. However, studying healthy does not only mean being free of physical and mental illness at the time of studying. Feeling healthy while studying also means, above all, feeling good while studying. In line with Ilmarinen's view, this includes above all the awareness that the construct of (health-related) study ability is multidimensional. There is much more to a healthy study than physical and mental integrity. In this case, the universally recognized WHO definition of health from 1946 should be cited again: “Health is a state of complete physical, mental and social well-being and not merely the absence of disease or infirmity” (9). The well-being mentioned in the WHO's definition of health is one of the most important things to aim for. A successful interaction between the health-related factors of the house of studyability (as in the floors of the house) paves the way to reach this aim. Without physical, mental, and social health, the factors of values, motivation and organization cannot be developed, even so, a flawed health-related value system or health-promoting working conditions imply that the functional capacities cannot be achieved. Figure 1 depicts the structural model and the interactions between the factors.

In the following work process, the data situation on the floors of the house of studyability is reviewed. With the involvement of the German Network Health Promoting Universities, the analysis can be extended to other universities. The analysis includes a research and review of previous empirically collected data in the following sub-areas of the studyability concept:

1. Competences, experience, learning: professional, methodical, and social supportive measures to cope with the tasks in the studies, such as writing homework, compiling a good timetable, observing the standard period of study, etc.,
2. Values, attitudes, and motivation: appreciation and fair treatment by lecturers, trust in lecturers, commitment, and motivation for studies.
3. Study, study community, and leadership: organization of studies, support by lecturers and fellow students in difficult situations, feedback from lecturers/teachers/examiners/administrators on administrative procedures (e.g., exam registration, grade entry).

Within this process, the house of studyability will be filled with data as well as instruments for the investigation of the mentioned topics. A systematization of the mentioned sub-areas is aimed at. The structure of the model is intended to ensure control of the application-oriented conception and implementation of interventions.

The result is a model that can be applied across institutions to develop a student health management at universities. The use of the future model structures can immediately be seen as a criterion for quality management.

The vision lies in the function of the model as:

- Heuristic model to classify already existing programs and measures at universities and thus identify missing structures and interventions.
- Instrument for the detection and identification of vulnerable groups (high risk exposure, low self-help), and/or need for change in organizational structures.
- Methodical manual for the structured development of a student health management system.
- Communication basis for scientific findings and the effectiveness of measures between health professionals and university management positions.

The aim is to ensure transfer of the measures and results to other sites and to ensure sustainable use with potential for generating added value for the establishment, implementation, and maintenance of a student health management at universities. With this model the appeal of the Okanagan Charter is being addressed. It intends to imply health promotion in universities with respect to the stated targets of the charter (10).

## Data Availability Statement

The original contributions presented in the study are included in the article/supplementary material, further inquiries can be directed to the corresponding author/s.

## Author Contributions

MK, JG, and KW wrote the manuscript. MK, JG, KW, and GH are working on the presented model. GH was the group leader of the project group. All authors contributed to the article and approved the submitted version.

## Conflict of Interest

The authors declare that the research was conducted in the absence of any commercial or financial relationships that could be construed as a potential conflict of interest.
